# Steroid profiling using liquid chromatography mass spectrometry during adrenal vein sampling in patients with primary bilateral macronodular adrenocortical hyperplasia

**DOI:** 10.3389/fendo.2022.1079508

**Published:** 2022-12-06

**Authors:** Ru Zhang, German Rubinstein, Sharmilee Vetrivel, Sonja Kunz, Frederick Vogel, Lucas Bouys, Jérôme Bertherat, Matthias Kroiss, Sinan Deniz, Andrea Osswald, Thomas Knösel, Martin Bidlingmaier, Silviu Sbiera, Martin Reincke, Anna Riester

**Affiliations:** ^1^ Medizinische Klinik und Poliklinik IV, LMU Klinikum, Ludwig-Maximilians-University, Munich, Germany; ^2^ Institut Cochin, Université Paris-Cité, Paris, France; ^3^ Department of Internal Medicine I, Division of Endocrinology and Diabetes, University Hospital, University of Würzburg, Würzburg, Germany; ^4^ Klinik und Poliklinik für Radiologie, LMU Klinikum, Ludwig-Maximilians-University, Munich, Germany; ^5^ Pathologisches Institut, Ludwig-Maximilians-University, Munich, Germany

**Keywords:** cortisol, AVS, steroidome, LC-MS/MS, adenoma, DHEA, reference hormone

## Abstract

**Introduction:**

Adrenal vein sampling (AVS) is not a routine procedure in patients with primary bilateral macronodular adrenocortical hyperplasia (PBMAH), but has been used to determine lateralization of cortisol secretion in order to guide decision of unilateral adrenalectomy. Our aim was to characterize the steroid fingerprints in AVS samples of patients with PBMAH and hypercortisolism and to identify a reference hormone for AVS interpretation.

**Method:**

Retrospectively, we included 17 patients with PBMAH from the German Cushing’s registry who underwent AVS. 15 steroids were quantified in AVS and peripheral blood samples using LC-MS/MS. We calculated lateralization indices and conversion ratios indicative of steroidogenic enzyme activity to elucidate differences between individual adrenal steroidomes and in steroidogenic pathways.

**Results:**

Adrenal volume was negatively correlated with peripheral cortisone (r=0.62, *p*<0.05). 24-hour urinary free cortisol correlated positively with peripheral androgens (rDHEA=0.57, rDHEAS=0.82, rA=0.73, rT=0.54, *p*<0.05). DHEA was found to be a powerful reference hormone with high selectivity index, which did not correlate with serume cortisol and has a short half-life. All investigated steroids showed lateralization in single patients indicating the heterogenous steroid secretion pattern in patients with PBMAH. The ratios of corticosterone/aldosterone (catalyzed by CYP11B2), androstenedione/dehydroepiandrosterone (catalyzed by HSD3B2) and cortisone/cortisol (catalyzed by HSD11B2) in adrenal vein samples were higher in smaller adrenals (*p*<0.05). *ARMC5* mutation carriers (n=6) showed lower androstenedione/17-hydroxyprogesterone and higher testosterone/androstenedione (*p*<0.05) ratios in peripheral blood, in line with lower peripheral androstenedione concentrations (*p*<0.05).

**Conclusion:**

Steroid profiling by LC-MS/MS led us to select DHEA as a candidate reference hormone for cortisol secretion. Lateralization and different steroid ratios showed that each steroid and all three steroidogenic pathways may be affected in PBMAH patients. In patients with germline *ARMC5* mutations, the androgen pathway was particularly dysregulated.

## Introduction

Primary bilateral macronodular adrenocortical hyperplasia (PBMAH) is a benign neoplastic disorder characterized by multiple nodules ≥ 10mm in diameter on both adrenals. The clinical presentation is variable, ranging from asymptomatic to overt symptoms of Cushing’s Syndrome (CS), and less commonly mineralocorticoid and/or androgens excess (1–3). This high heterogeneity and lack of specific symptoms renders PBMAH difficult to identify, and criteria for medical treatment or adrenalectomy still remain to be established. Given the complex pathophysiology of PBMAH, analyses of individual adrenal steroidome and comparison of interadrenal differences in steroidogenesis in patients with PBMAH may be of value to better characterize biochemical features and the steroid pathways involved. However, to the best of our knowledge, comprehensive data on steroid fingerprinting of the effluent of adrenal veins, and correlation between steroid patterns and clinical parameters have not yet been published.

Adrenal vein sampling (AVS) is the gold standard used to distinguish unilateral from bilateral forms of primary aldosteronism (PA). Patients with unilateral PA are usually referred to surgery. In selected patients with PBMAH, unilateral adrenalectomy can be a therapeutic approach despite bilateral disease (4–7). This could have the advantage over bilateral adrenalectomy of decreasing the risk for life-threatening adrenal crises and obviate the lifelong adrenocortical hormone replacement (8, 9). Previous studies addressed the application of AVS in PBMAH with CS to guide unilateral adrenalectomy (10–12). However, the variations of AVS protocols in use have been a limiting factor so far, and no consensus on a reference hormone has been achieved, which is required for diagnostic selectivity and to account for sample dilution. Therefore, a reliable reference hormone is necessary for improved interpretation of AVS results. Liquid chromatography-mass spectrometry (LC-MS/MS) for multiple steroids measurements in AVS samples enables a comprehensive appraisal of adrenal steroid output. Calculation of product/precursor ratios provides insights into adrenal steroidogenesis through analysis of the activity of different steroidogenic enzymes and thus allows investigations into alterations of steroidogenic pathways in the adrenal hyperplasia (13).

We hypothesized - based on variability of histopathologic phenotypes - that steroid secretion in PBMAH might be heterogeneous, with differences in steroid fingerprints between individual patients and even between the adrenal glands of the same patient.

To confirm our hypotheses, we followed these steps (1): correlation of steroid profiles with clinical parameters (2); identification of a reference hormone for AVS interpretation in patients with PBMAH using LC-MS/MS; (3) investigation of alterations in inter-adrenal steroidome using lateralization index (LI) and steroidogenic pathways using conversion analysis (product/precursor) in AVS samples.

## Methods

### Subjects

For this retrospective analysis, we included 17 patients with PBMAH from the German Cushing’s registry who underwent AVS between 2006 to 2021 (15 treated at the LMU Klinikum, Ludwig Maximilians University Munich, Germany and 2 at the University Hospital of Würzburg, Julius Maximilians University Würzburg, Germany). These patients had documented ACTH-independent Cushing’s syndrome and bilateral adrenal masses typical for PBMAH. AVS was performed to identify a hormonally dominant side of cortisol production to guide unilateral adrenalectomy. Most of these patients (patients 1 to 12, patient 17 and patient 18) were already part of our study on the clinical role of AVS in PBMAH (14). As described also in that publication, patients underwent biochemical screening for Cushing’s syndrome by the three recommended screening tests: 1-mg dexamethasone suppression test (LDDST), late-night salivary cortisol (LNSLC) and 24h urinary free cortisol (UFC), all performed using immunoassay. Germline *ARMC5* (armadillo repeat containing 5) sequencing was performed in 15 patients. Inactivating mutations of *ARMC5*, a putative tumor-suppressor gene, are associated with a more severe hypercortisolism, bigger adrenals with a higher number of nodules (15).The assays for the screening tests, baseline ACTH and *ARMC5* status were described in the online Supplementary Material (14).

The study was approved by the LMU ethics committee (Project number: 152-10) and performed in accordance with the principles of the declaration of Helsinki. All participants gave written informed consent.

### Adrenal vein sampling

The decision to perform AVS for patients with bilateral adrenal masses was made independently by the treating endocrinologist before 2012 or by a multidisciplinary endocrine board since 2012. Our group described in detail the exact procedure previously (14). AVS was conducted without ACTH stimulation and peripheral blood samples were collected simultaneously with each of the selective blood samples. Samples were stored at -80°C until analysis. As no guideline is available for AVS performed in PBMAH patients, we interpreted the results in analogy to the Endocrine Society Practice Guideline on primary aldosteronism (PA) (16). Successful catheterization was confirmed in an exploratory analysis by a gradient of steroid concentrations between adrenal vein to peripheral vein (AV/PV, also called selectivity index, SI) greater than 2 (16–18). To analyze lateralization of hormone production we calculated a lateralization index, which is defined by the ratio of the high side to the low side of the corrected steroid of interestlevels (17). Following this definition, LI in our study is defined as:


$$\text{LI}=\frac{\left(\frac{steroid}{ipsilateral\;reference\;hormone}\right)high\;side\;}{\left(\frac{steroid}{ipsilateral\;refrence\;hormone}\right)low\;side}$$

This formula can be used for any steroid of interest, as long as the reference steroid in the denominator position is not affected by the rate of secretion of the steroid in the numerator position. In our PBMAH patient cortisol was used as steroid of interest. Recommendation for unilateral or bilateral adrenalectomy was not based on the results of LC-MS/MS presented in this study. The decision of the interdisciplinary tumour board was guided instead by the severity of CS (clinically and biochemically), the results of radiologic studies, plus AVS results measured with immunoassay as described in our previous study (14).

### Steroids analysis by LC-MS/MS

A panel of 15 steroids was quantified in archival AVS and peripheral EDTA-plasma samples using the commercially available MassChrom^®^ Steroid LC-MS/MS kit (Chromsystems, Gräfelfing, Germany) and a 1290 Infinity II ultra-high performance liquid chromatography instrument (Agilent Technologies, Santa Clara, USA) connected to a QTRAP6500+ triple quadrupole mass spectrometer (ABSciex, Framingham, USA). Sample preparation was performed *via* offline solid phase extraction of 500µL sample according to the instructions of the manufacturer. Before extraction, the respective stable isotope labeled steroids were added as internal standards. Twenty microliters were injected to the LC-MS/MS system, ionized with electrospray ionization (ESI) and analyzed in multiple reaction monitoring mode. Aldosterone and DHEA-S were measured in negative ESI mode. All other steroids were measured in positive ESI mode. A six-point calibration with 1/x^2^ weighting was used for quantification of the steroids by the SciexOS software (Version 1.6.1, ABSciex, Framingham, USA). Sample containing steroid concentrations above the highest calibration were re-assayed after dilution in 0.9% saline, and results multiplied by the dilution factor. Quality control samples provided by the manufacturer were measured within each analytical run to continuously monitor performance of the LC-MS/MS measurements. We regularly participated in the national external quality assessment scheme for steroid hormones (Reference Institute for Bioanalytics, RfB, Bonn, Germany) and passed for all included steroids. The kit includes the following steroids: progesterone (Prog), 17-hydroxyprogesterone (17OHP), cortisol (F), 11-deoxycortisol (11dF), 21-deoxycortisol(21dF), cortisone (E), corticosterone (Cort), 11-deoxycorticosterone (DOC), aldosterone (Aldo), dehydroepiandrosterone (DHEA), dehydroepiandrosterone sulfate (DHEAS), dihydrotestosterone (DHT), testosterone (T), androstenedione (A) and estradiol (E2). The low limit of quantification (LLOQ) and up limit of quantification (ULOQ) for each steroid are summarized in **Supplemental Data** (**Table S1**). To study the alteration of adrenal steroidogenesis pathways, conversion ratios based on adrenal size and radiological asymmetry were calculated with adrenal vein metabolite/its precursor, and conversion ratios based on *ARMC5* status calculated with peripheral metabolite/its precursor, accordingly (19, 20).

### Adrenal size

The adrenal volume was measured in three dimensions and calculated by height x width x depth. One patient provided only axial planes. Therefore, in this patient adrenal size was assessed by the maximum diameter in cm. The definition of adrenal asymmetry was a difference > 30% between adrenal volumes or, if not available, the maximum diameter.

### Statistics

We analyzed correlation using a two-tailed Spearman correlation coefficient. Mann Whitney test was used to assess the differences of peripheral steroids and clinical parameters based on *ARMC5* status, and conversion ratios based on adrenal size and *ARMC5* status. The differences of conversion ratios between groups based on radiological asymmetry were evaluated by Wilcoxon test. Steroid concentrations below LLOQ and without peak were calculated as 0.5*LLOQ. A value of *p*<0.05 was considered statistically significant. We used Microsoft Excel 365 for data calculation and GraphPad Prism 8 for the statistical analyses.

## Results

### Correlation of clinical parameters and peripheral steroidome

16/17 patients were females, 15 patients were older than 55 years (see **Supplementary Table S1**). The biochemical evaluation demonstrated the typical features of adrenal hypercortisolism. *ARMC5* status was evaluated in 15 patients. 6 patients showed mutations in the *ARMC5* gene, while 9 patients had the wildtype.

**Figure 1:**
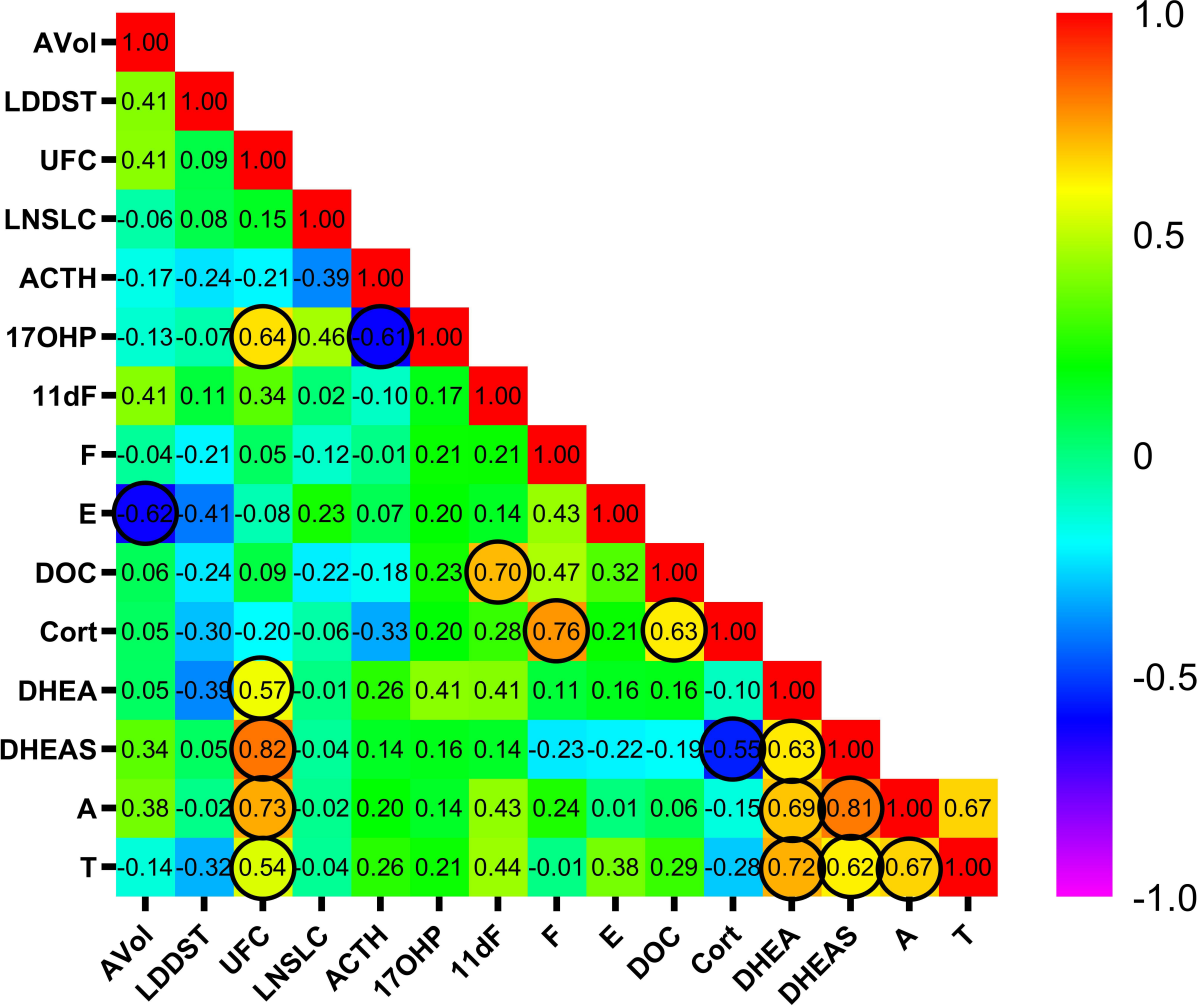
Correlation between adrenal volume, the three diagnostic tests of hypercortisolism, and peripheral steroid concentrations as measured by LC-LC/MS. The values in cells are the coefficient r, the circles indicate statistical significance (*p*<0.05). Male and premenopausal female patients were excluded. AVol: adrenal volume; UFC, 24h urinary free cortisol; LDDST, low dose dexamethasone suppression test; LNSLC, late-night salivary cortisol; late-night salivary cortisol; ACTH, adrenocorticotropic hormone. 17OHP, 17-hydroxyprogesterone; 11dF, 11-deoxycortisol; F, cortisol; E, cortisone; DOC, 11-deoxycorticosterone; Cort, corticosterone; DHEA, dehydroepiandrosterone; DHEAS, dehydroepiandrosterone sulfate; A, androstenedione; T, testosterone.

Correlations between adrenal volume, baseline ACTH, the three diagnostic tests for CS and the peripheral steroids were analyzed in 15 postmenopausal patients (**Figure 1**); the male (patient 4) and one premenopausal female (patient 6) were excluded from this analysis. The concentrations of aldosterone (7 patients), 21-deoxycortisol (4 patients), estradiol (8 patients), DHT (8 patients), 11-deoxycorticosterone (2 patients) and progesterone (8 patients) were below LLOQ or detection in >10 % of the samples (**Supplementary Table S2**). Therefore, we excluded these steroids from further analysis. Adrenal volume showed negative correlations with plasma cortisone concentrations (r=-0.62, *p*<0.05). UFC did not correlate with plasma cortisol but did correlate positively with 17-hydroxyprogesterone (r=0.64, *p*<0.05), DHEA (r=0.57, *p<*0.05), DHEAS (r=0.82, *p<*0.001), androstenedione (r=0.73, *p<*0.01) and testosterone (r=0.54, *p<*0.05) in peripheral plasma samples. The association of steroid concentrations with germline *ARMC5* mutation status is presented in **Table 1**. Patients with germline *ARMC5* mutations had lower plasma androstenedione concentrations than wild-type patients.

**Table 1:** Comparison of peripheral steroids (ng/ml) based on *ARMC5* status.

Peripheral steroids	Wild-type *ARMC5 *(n=9)	Mutant *ARMC5 *(n=6)	*p* value
**F**	117.2 [92.8, 153.5]	119.6 [83.0, 163.8]	0.77
**E**	16.1 [14.1, 17.7]	13.9 [12.3, 18.4]	0.55
**Cort**	1.8 [0.8, 3.7]	2.5 [1.6, 17.8]	0.18
**11dF**	0.97 [0.45, 1.19]	0.48 [0.35, 1.04]	0.53
**DHEAS**	314.0 [221.0, 795.4]	119.0 [45.4, 284.1]	0.06
**T**	0.13 [0.07, 0.20]	0.06 [0.04, 0.15]	0.18
**A**	0.77 [0.32, 1.50]	0.27 [0.13, 0.51]	<0.05
**DHEA**	0.85 [0.33, 1.66]	0.48 [0.13, 0.67]	0.13
**17OHP**	0.19 [0.15, 0.28]	0.31 [0.12, 0.47]	0.53
**DOC**	0.07 [0.04, 0.12]	0.04 [0.04, 0.31]	0.75

Data are presented with median [Q1, Q3]. F, cortisol; 11dF, 11-deoxycortisol; 17OHP, 17-hydroxyprogesterone; E, cortisone; DOC, 11-deoxycorticosterone; Cort, corticosterone; DHEA, dehydroepiandrosterone; DHEAS, dehydroepiandrosterone sulfate; A, androstenedione; T, testosterone.

### Identification of a reference hormone

An ideal reference hormone to be used during AVS for correction of dilution effects in adrenal veins should have a rather short half-life and should be secreted independently of the underlying adrenal pathology. Therefore, the selection of the best reference hormone was based on three steps in our study (1): The candidate hormone should show a concentration gradient between adrenal and peripheral vein (selectivity index) ≥ 2 (2); The candidate hormone should not correlate with cortisol, which would indicate a co-secretion by the cortisol-producing tumor (3); The candidate hormone has a short half-time.

**Figure 2:**
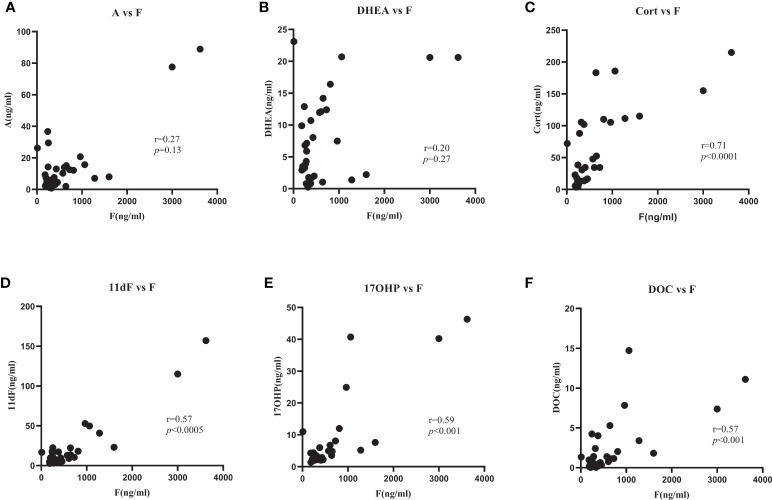
Correlation of cortisol vs six steroids with high gradient of concentration from adrenal vein to peripheral vein: androstendione (**A**), DHEA (**B**), corticosterone (**C**), 11-deoxycortisol (**D**), 17-hydroxyprogesteron (**E**), 11-deoxycorticosterone (**F**). F, cortisol; A, androstenedione; DHEA, dehydroepiandrosterone; Cort, corticosterone; 11dF, 11-deoxycortisol; 17OHP, 17-hydroxyprogesterone; DOC, 11-deoxycorticosterone.

In order to evaluate specific steroids as reference hormones, a ratio of adrenal vein/peripheral vein called selectivity index (SI) was calculated for each steroid. SI > 2 was selected as the traditional cutoff to assess the success of AVS cannulation (16–18), in analogy to AVS for primary aldosteronism (**Table 2**). In the right AVS of patient 17, only aldosterone showed an SI > 2. We therefore concluded a failure of cannulation of the right adrenal vein of patient 17, and this sample was excluded from further analysis. 17-hydroxyprogesterone, 11-deoxycortisol, corticosterone, DHEA, androstenedione and 11-deoxycorticosterone indicated successful catheterization in >90% of the cases. Thus, correlation analyses between these six steroids and cortisol were performed (**Figure 2**). Corticosterone (r=0.71, *p*<0.0001), 11-deoxycortisol (r=0.57, *p*<0.0005), 17-hydroxyprogesterone (r=0.59, *p*<0.001) and 11-deoxycorticosterone (r=0.57, *p*<0.001) showed significant correlations with cortisol, indicating a co-secretion with the cortisol-producing tumor. Androstenedione (r=0.27, *p*=0.13) and DHEA (r=0.20, *p*=0.27) had no correlation with cortisol. Compared with androstenedione (*T1/2*_A_ is about 30 min) (21), DHEA (*T1/2*_DHEA_ is 15 to 30 min) (22, 23) has a shorter half-life. As a result, we concluded that DHEA could be a viable reference hormone, meeting all the criteria we defined previously.

**Table 2:** Concentration gradient (selectivity index, SI) of each steroid from adrenal vein (AV) to peripheral vein (PV) in 17 patients.

Steroids	Successful catheterization*	AV/PV	1	2	3	4	5	6	7	8	9	10	11	12	13	14	15	16	17
Prog	13/17	LAV/PV	6.67	376.74	7.53	18.58	10.06	12.46	3.90	1.56	1047.62	6.47	18.26	1.00	19.96	1.00	23.01	1.60	29.36
	12/17	RAV/PV	4.76	20.23	6.59	22.08	48.39	17.67	19.74	1.62	1841.27	999.75	1.00	1.00	10.90	1.00	30.20	1.40	1.00
**17OHP**	17/17	LAV/PV	14.22	166.00	11.60	5.12	12.30	162.75	9.83	18.86	46.25	14.34	17.54	8.58	14.14	21.18	25.27	18.26	80.20
	16/17	RAV/PV	9.74	19.13	12.61	8.68	34.48	187.45	38.46	5.04	38.13	162.19	5.25	15.88	9.86	8.80	25.97	19.01	1.90
21dF	13/17	LAV/PV	14.91	0.64	8.13	0.09	2.60	90.11	2.05	47.01	3.47	4.11	19.27	1.00	6.66	8.49	3.70	1.89	9.58
	14/17	RAV/PV	12.50	2.69	6.07	4.27	11.90	123.63	10.73	15.58	4.95	34.88	5.98	1.00	4.20	3.09	1.00	5.00	0.81
**11dF**	17/17	LAV/PV	13.56	40.62	31.48	12.69	4.58	112.75	13.39	58.08	54.77	11.65	16.07	10.20	9.41	24.27	22.19	13.26	36.08
	16/17	RAV/PV	17.05	7.42	27.34	9.89	16.76	153.92	57.79	12.45	16.38	79.81	5.69	16.50	7.22	10.14	21.48	10.59	1.44
F	16/17	LAV/PV	4.05	5.29	3.05	3.81	1.14	27.52	3.75	15.38	9.48	2.49	3.73	2.17	2.35	3.13	3.99	3.87	9.78
	12/17	RAV/PV	4.31	2.36	1.88	2.24	5.13	33.21	0.10	3.88	1.81	9.04	1.63	2.65	2.07	2.00	3.50	2.51	0.96
E	10/17	LAV/PV	0.83	1.67	3.65	2.80	1.15	6.82	1.34	5.23	2.55	1.89	2.41	0.89	2.04	1.44	2.53	2.30	5.27
	7/17	RAV/PV	0.97	1.39	3.20	3.26	1.72	6.89	3.11	2.14	0.96	3.87	1.45	0.74	1.94	1.17	2.01	1.55	0.93
**DOC**	16/17	LAV/PV	10.76	75.38	84.50	11.97	7.32	107.28	3.80	41.74	23.38	10.75	17.10	8.96	9.06	10.65	15.45	1.43	33.14
	15/17	RAV/PV	12.33	6.17	68.50	9.10	15.11	161.57	22.50	12.91	29.24	358.06	5.77	10.68	5.30	4.54	19.13	1.29	1.00
**Cort**	17/17	LAV/PV	16.34	20.43	8.55	10.78	2.49	101.97	10.88	56.65	48.02	7.45	13.45	9.04	5.21	16.17	11.56	12.97	72.42
	16/17	RAV/PV	13.99	2.97	6.37	6.00	12.00	141.45	57.68	16.95	16.55	103.61	3.44	10.49	4.35	6.79	10.52	7.67	1.39
Aldo	14/17	LAV/PV	8.60	29.55	0.14	0.16	524.14	38.98	–	36.97	–	0.88	27.91	11.83	6.88	5.82	6.01	6.94	81.42
	15/17	RAV/PV	7.35	5.45	3.15	0.16	0.38	46.34	–	16.68	–	7.99	5.41	14.52	17.05	5.74	6.01	32.20	6.78
**DHEA**	17/17	LAV/PV	7.09	8.66	7.77	2.13	2.62	63.38	10.89	4.59	11.39	6.09	17.09	4.12	2.36	10.50	9.99	11.07	19.38
	15/17	RAV/PV	3.74	9.31	16.84	1.69	4.34	63.38	23.50	3.64	29.84	17.66	5.45	3.89	7.46	5.20	8.42	19.62	1.31
DHEAS	0/17	LAV/PV	0.82	1.05	1.19	1.16	1.02	1.95	1.20	0.97	1.03	1.08	1.48	1.25	1.17	1.23	1.30	1.00	1.42
	0/17	RAV/PV	1.10	1.09	1.38	1.11	1.22	1.89	1.32	1.26	1.00	1.33	1.13	1.00	1.11	1.07	1.14	1.00	0.87
**A**	17/17	LAV/PV	22.11	9.28	5.91	6.76	11.83	159.02	15.17	29.16	25.80	15.26	20.57	7.27	10.53	18.81	25.82	21.80	37.20
	16/17	RAV/PV	17.77	5.83	6.38	10.51	15.41	182.17	52.51	11.17	20.22	46.89	6.01	14.15	14.39	7.32	17.53	26.63	1.24
T	8/17	LAV/PV	1.72	2.46	2.04	0.77	1.62	4.67	1.30	2.62	1.53	1.41	1.84	2.07	0.80	2.14	1.94	2.46	2.62
	5/17	RAV/PV	1.44	1.83	2.82	0.72	3.38	8.22	2.26	1.46	1.30	3.91	1.08	1.53	0.96	1.00	1.95	1.72	1.48
DHT	1/17	LAV/PV	0.74	0.64	2.21	1.19	1.00	1.21	4.86	0.15	0.95	1.00	1.00	1.00	1.00	1.00	1.00	1.00	1.00
	0/17	RAV/PV	1.01	1.12	1.30	1.12	1.00	1.10	1.04	0.15	1.37	1.00	1.00	1.00	1.00	1.00	1.00	1.00	1.00
E2	2/17	LAV/PV	1.35	0.16	1.29	0.94	0.06	0.58	1.61	0.75	2.63	1.00	1.00	1.00	1.00	1.95	7.83	1.00	1.00
	3/17	RAV/PV	1.69	0.08	1.08	0.87	0.30	0.18	4.76	0.94	9.57	1.00	1.00	13.79	1.00	0.30	0.35	1.00	1.00

*Number of samples with successful catheterization (selectivity index>2); AV, adrenal vein; PV, peripheral vein; LAV, left adrenal vein; RAV, right adrenal vein; Prog, progesterone; 17OHP, 17-hydroxyprogesterone; 21dF, 21-deoxycprtisol; 11dF, 11-deoxycortisol; F, cortisol; E, cortisone; DOC, 11-deoxycorticosterone; Cort, corticosterone; Aldo, aldosterone; DHEA, dehydroepiandrosterone; DHEAS, dehydroepiandrosterone sulfate; A, androstenedione; T, testosterone; DHT, dihydrotestosterone; E2, estradiol. The six steroids with the highest SI values are marked in bold.

### Comparison of the steroidome between both adrenals of individual patient and steroidogenic pathways analysis

**Figure 3:**
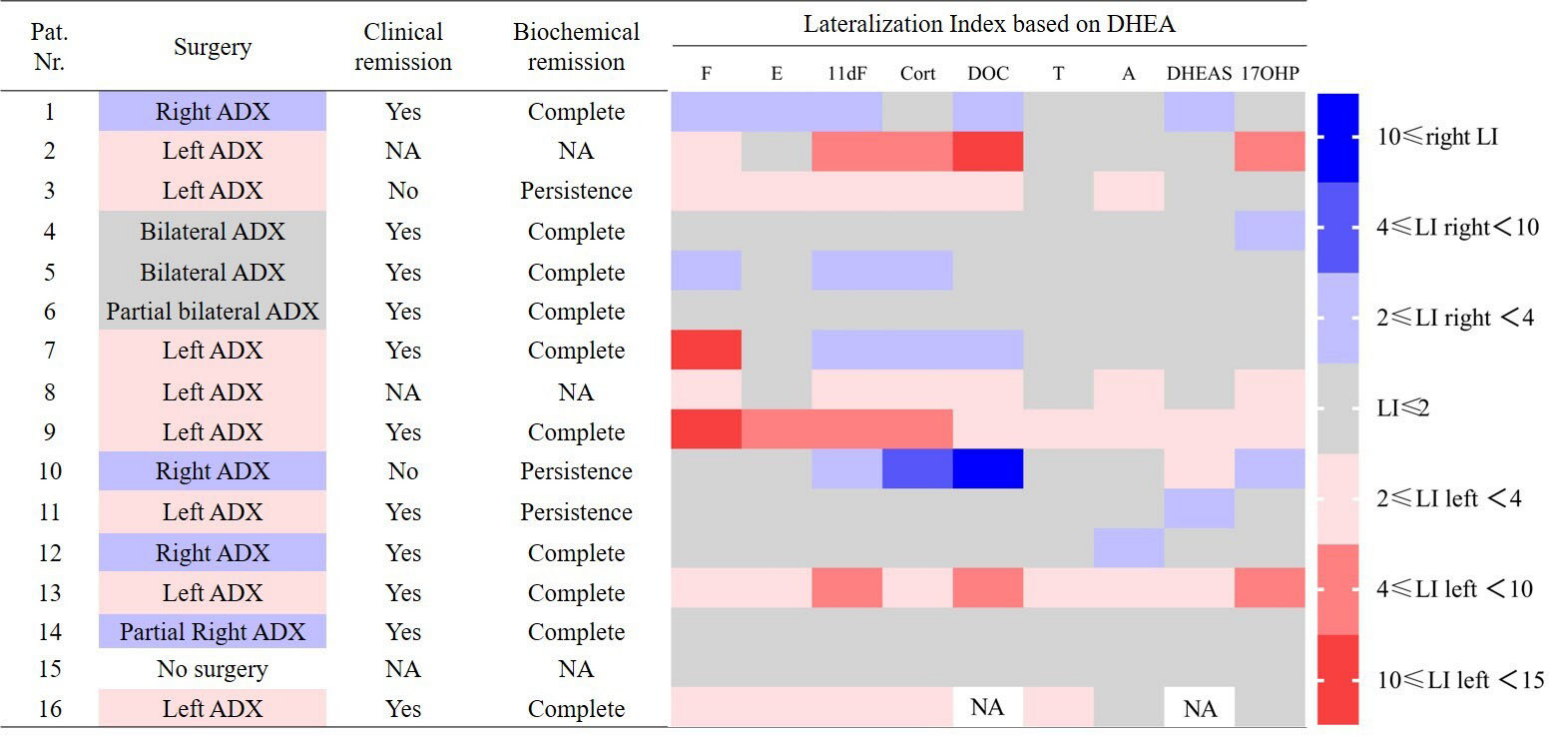
Comparison between lateralization index (LI), surgery and outcome in patients with PBMAH. Steroids below LLOQ or dectection were excluded in the figure. Pat. No. 17 was excluded because of failed adrenal vein cannulation. NA, not available; ADX, adrenalectomy; F, cortisol; E, cortisone; 11dF, 11-deoxycortisol; Cort, corticosterone; DOC, 11-deoxycorticosterone; T, testosterone; A, androstenedione; 17OHP, 17-hydroxyprogesterone.

We calculated the lateralization index to investigate differences in steroid fingerprints between the adrenals of each patient. **Figure 3** shows the lateralization indices of the nine steroids, which could be measured adequately, using DHEA as reference hormone. The results indicate that the secretion of all measured steroids could be affected in PBMAH: every measured steroid showed a lateralization (LI above 2) in at least one patient. On the other hand, not every patient with PBMAH showed a lateralization of steroid production: 3 patients (patient 6, 14 and 15) had no lateralization at all when lateralization was defined as LI above 2. If defining LI above 4 as a relevant lateralization in steroid production, only 5 patients fulfilled this criterion by at least one steroid. However, out of these, in two patients (patient 7 and patient 10) the dominant side was inconsistent: In patient 7, the secretion of cortisol was mainly left (LI=82.7), while 11-deoxycortisol (LI=2.0), corticosterone (LI=2.5) and 11-deoxycorticosterone (LI=2.7) were mainly produced by the right adrenal. In patient 10, DHEAS (LI=2.4) production lateralized to the left side, whereas 11-deoxycortisol (LI=2.4), corticosterone (LI=4.8), and 11-deoxycorticosterone (LI=11.5) lateralized to the right side.

**Figure 4:**
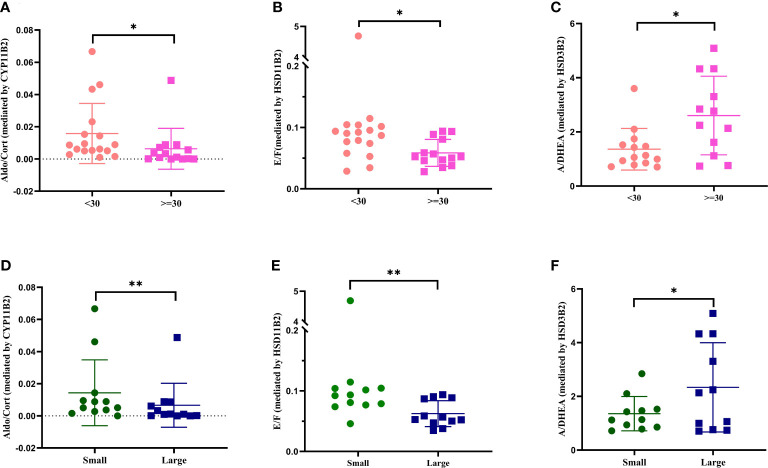
Conversion ratio analysis of adrenal vein values based on adrenal volume and radiological asymmetry. Only significant results are shown. Comparisons of Aldo/Cort between groups based on adrenal volume **(A)** and radiological asymmetry **(D)**. Comparison of E/F between groups based on adrenal volume **(B)** and radiological asymmetry **(E)**. Comparison of A/DHEA between groups based on adrenal volume **(C)** and radiological asymmetry **(F)**. Aldo, aldosterone; Cort, corticosterone; A, androstenedione; DHEA, dehydroepiandrosterone; E, cortisone; F, cortisol. *p<0.05, **p<0.01

Additionally, conversion ratios (steroid/precursor) based on adrenal size and on *ARMC5* status values were calculated to identify possible differences in affected steroid pathways. All conversion ratios among the pathway of adrenal steroidogenesis were analyzed (**Supplementary data, Figure S1**). For conversion ratios involving sex hormones, only the values of the postmenopausal women were included. 14 adrenals had a volume ≥ 30 ml, 18 adrenals had a volume< 30 ml, 12 patients showed radiological asymmetry. Aldosterone/corticosterone and cortisone/cortisol were higher in the group of adrenals with volumes< 30 ml (**Figures 4A, B**) and radiologically small adrenals (**Figures 4D, E**). Androstenedione/dehydroepiandrosterone was higher in the group of adrenals with volumes ≥ 30 ml (**Figure 4C**) and radiologically large adrenals (**Figure 4F**).

**Figure 5:**
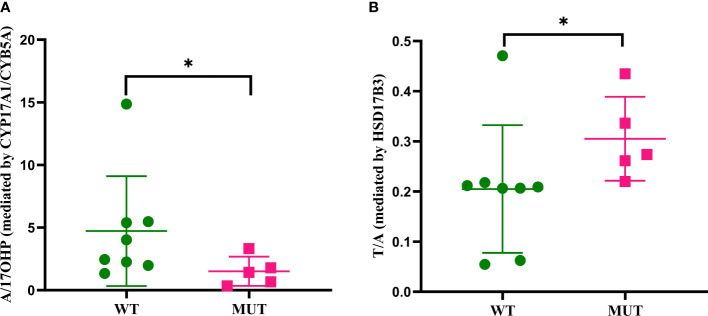
Conversion ratio analysis of peripheral steroids based on *ARMC5* status. Only significant conversion ratios are displayed. **(A)** Comparison of A/17OHP between wild-type *ARMC5* patients and mutant *ARMC5* patients. **(B)** Comparison of T/A between wild-type *ARMC5* patients and mutant *ARMC5* patients. A, androstenedione; 17OHP, 17-hydroxyprogesterone; T, testosterone. WT, wild-type *ARMC*5 patients; MUT, mutant *ARMC5* patients. *p<0.05

*ARMC5* mutation carriers showed lower conversion ratios of androstenedione/17-hydroxyprogesterone and higher testosterone/androstenedione than *ARMC5*-wildtype patients (*p*<0.05, **Figures 5A, B**).

## Discussion

We performed an exploratory study in patients with PBMAH using LC-MS/MS measurement in peripheral blood and adrenal vein samples to investigate correlations among clinical parameters and peripheral steroid concentrations, to identify a potential reference hormone, and to compare the steroidome between both adrenals of individual patients and analyze affected steroidogenic pathways.

### Correlations among clinical parameters and peripheral steroids in PBMAH

No correlation between adrenal volume and peripheral cortisol level was found. Even though UFC better reflects the 24-hour time-integrated cortisol secretion compared to random serum cortisol, still no correlation between adrenal volume and UFC was observed indicating variable steroidogenic efficiency between PBMAH cases. This is in line with the findings of Wurth et al. in a cohort of 44 patients with PBMAH, in which adrenal volume was calculated based on computed tomography scans (24). 17-hydroxyprogesterone, DHEA, DHEAS, androstenedione and testosterone showed a positive correlation with UFC. In contrary, no correlation or even negative correlation (DHEAS) with serum cortisol was seen. Also, serum cortisol was not correlated with UFC. A possible explanation could be, that cortisol secretion levels vary throughout the day and UFC reflects the daily cortisol output. As a genetic disease, PBMAH is reported to be associated with germline mutation in *ARMC5* gene. Mutation carriers are found to have an more severe Cushing phenotype than patients with wild-type *ARMC5* (25). In the cohort described by Espiard et al., germline *ARMC5*-mutation is associated with lower ACTH, and higher UFC and higher cortisol after dexamethasone suppression test compared to wild-type PBMAH patients (26). This was not observed in our cohort (**Supplementary Table S3**), probably due to the relatively small cohort size.

### Selection of reference hormone for AVS interpretation

At the moment cortisol is commonly used as a reference hormone to correct for dilution effects during AVS for PA. According to the experience of AVS in PA, a successful adrenal vein cannulation is traditionally defined by a SI >2 when AVS is performed without ACTH stimulation (16–18). In PA, the selection of cortisol as reference hormone is based on the assumption that cortisol is entirely secreted by the normal zona fasciculata and not overproduced by the aldosterone-producing lesion, which however has limitations in case of pronounced aldosterone and cortisol co-secretion (17, 27). Metabolites with long half-life are slowly cleared from circulation, and have decreasing adrenal to peripheral gradients, thus, this will impair the interpretation of AVS results. As a result, we selected the reference hormone based on a three-step approach accounting for these factors. According to our findings, we identified DHEA as the most promising reference hormone in patients with PBMAH.

### Alterations in interadrenal steroidome and steroidogenic pathways in PBMAH

AVS was firstly introduced to guide surgical decision making in ACTH-independent Cushing’s syndrome by colleagues from the Mayo Clinic (5). In the following years, AVS was performed occasionally in adrenal CS by several centers. Due to the limited sample size and outcome data, our study was not performed to evaluate the optimal LI cut off, rather, the study intended to evaluate the LI of all steroids to evaluate adrenal laterality in patients with PBMAH. Our results nicely illustrate that cortisol lateralization based on AVS is valuable in most patients with PBMAH. Interestingly, corticosterone and 11-deoxycortisol showed also pronounced lateralization effects. So taken together one can say that cortisol is one of the most dysregulated hormones in PBMAH pathology, but all other steroids could be affected, too.

Lower conversion ratios of cortisone/cortisol (catalyzed by HSD11B2) and aldosterone/corticosterone (catalyzed by aldosterone synthase, CYP11B2) were observed in larger adrenals both based on adrenal volumes and radiological asymmetry. HSD11B2 is not expressed in normal adrenals (28), but expressed in adrenal adenomas (28, 29). Therefore, this result is unexpected. However, up to our knowledge the expression status of HSD11B2 in adrenals of PBMAH patients have not been investigated yet.CYP11B2 was undetectable by immunohistochemistry in the tumor of adrenal CS in a study by Nishimoto et al. (30). Similar immunohistochemical analyses have not yet been done in PBMAH, but it could be speculated that lower synthesis of aldosterone in adrenals affected by PBMAH is possibly caused by CYP11B2 repression. Higher conversion ratio of androstenedione/dehydroepiandrosterone (catalyzed by CYP17A1 or CYB5A) in larger adrenals indicates possible dysregulation in androgenic steroids in PBMAH. Taken together, even though PBMAH is primarily associated with cortisol excess, there are co-secretion of steroids of the mineralocorticoid and androgen pathways. In addition, we observed patients with germline *ARMC5* variants have lower androstenedione/17-hydroxyprogesterone and higher testosterone/androstenedione conversion ratios. In line with these findings, peripheral androstenedione concentrations in germline *ARMC5* mutation carriers were decreased, indicating that the androgen steroid pathway is dysregulated in PBMAH patients carrying a germline *ARMC5* mutation, possibly through decreased ACTH. In line with this notion, lower peripheral DHEAS levels measured by LC-MS/MS in germline *ARMC5* mutation carriers were described in another cohort (31). We saw the same tendency in our cohort, but this finding failed to be significant (119 vs 314 ng/ml, p=0.06).

## Conclusion

In summary, our study showed some distinct correlations between the adrenal volume, baseline ACTH, the three diagnostic tests for hypercortisolism (LDDST, LNSLC and UFC) and circulating steroids of PBMAH. If AVS is performed in patients with PBMAH, DHEA could be used as reference hormone. Comparative analyses of steroids by LC-MS/MS identified different steroid fingerprints among PBMAH patients and emphasize the heterogeneity of this disease. Germline mutations in the *ARMC5* gene were found to affect the androgen pathway in particular.

## Limitations

There are some limitations of our study. (1) The synthesis of sex hormones is affected by gender and age. Due to the limited sample size and biased sex constitution, we were unable to study the correlations between clinical parameters and peripheral steroids based on gender and age. (2) Some patients were lost to follow up after treatment. Therefore, the recurrence of hypercortisolism was not evaluated. (3) AVS is invasive so that having the AVS data from healthy people as control is impossible. (4) Our data is descriptive and further research are needed to confirm our results.

## Data availability statement

The original contributions presented in the study are included in the article/**Supplementary Material**. Further inquiries can be directed to the corresponding author.

## Ethics statement

The studies involving human participants were reviewed and approved by LMU ethics committee. The patients/participants provided their written informed consent to participate in this study.

## Author contributions

RZ and GR contributed equally to this work and share first authorship. RZ, GR, MR, and AR conceived and planned the experiments. GR, FV, MK, AO, and TK collected the data and provided samples. RZ, GR, SK, LB, JB, and SD performed the experiments. SV, JB, MK, MB, SS, MR and AR contributed to the interpretation of the results. RZ, GR and AR wrote the manuscript. All authors provided critical feedback. All authors contributed to the article and approved the submitted version.
