# Supplementary material for: Steroid profiling using liquid chromatography mass spectrometry during adrenal vein sampling in patients with primary bilateral macronodular adrenocortical hyperplasia
Source: Front Endocrinol (Lausanne). 2022 Dec 6;13:1079508. doi: 10.3389/fendo.2022.1079508 (PMC9763872; doi:10.3389/fendo.2022.1079508)
Supplement: Supplementary file 1 [file DataSheet_1.pdf]

Supplementary data from:

Steroid profiling using liquid chromatography mass spectrometry during adrenal vein sampling in patients with primary bilateral macronodular adrenocortical hyperplasia (PBMAH)

Ru Zhang†, German Rubinstein†, Sharmilee Vetrivel, Sonja Kunz, Frederick Vogel, Lucas Bouys, Jérôme Bertherat, Matthias Kroiss, Sinan Deniz, Andrea Osswald, Thomas Knösel, Martin Bidlingmaier, Silviu Sbiera, Martin Reincke, Anna Riester*

The associated article has been submitted to the *Frontiers in Endocrinology*. Please cite the original article when using these data.

Supplementary Material

1 Supplementary Figures

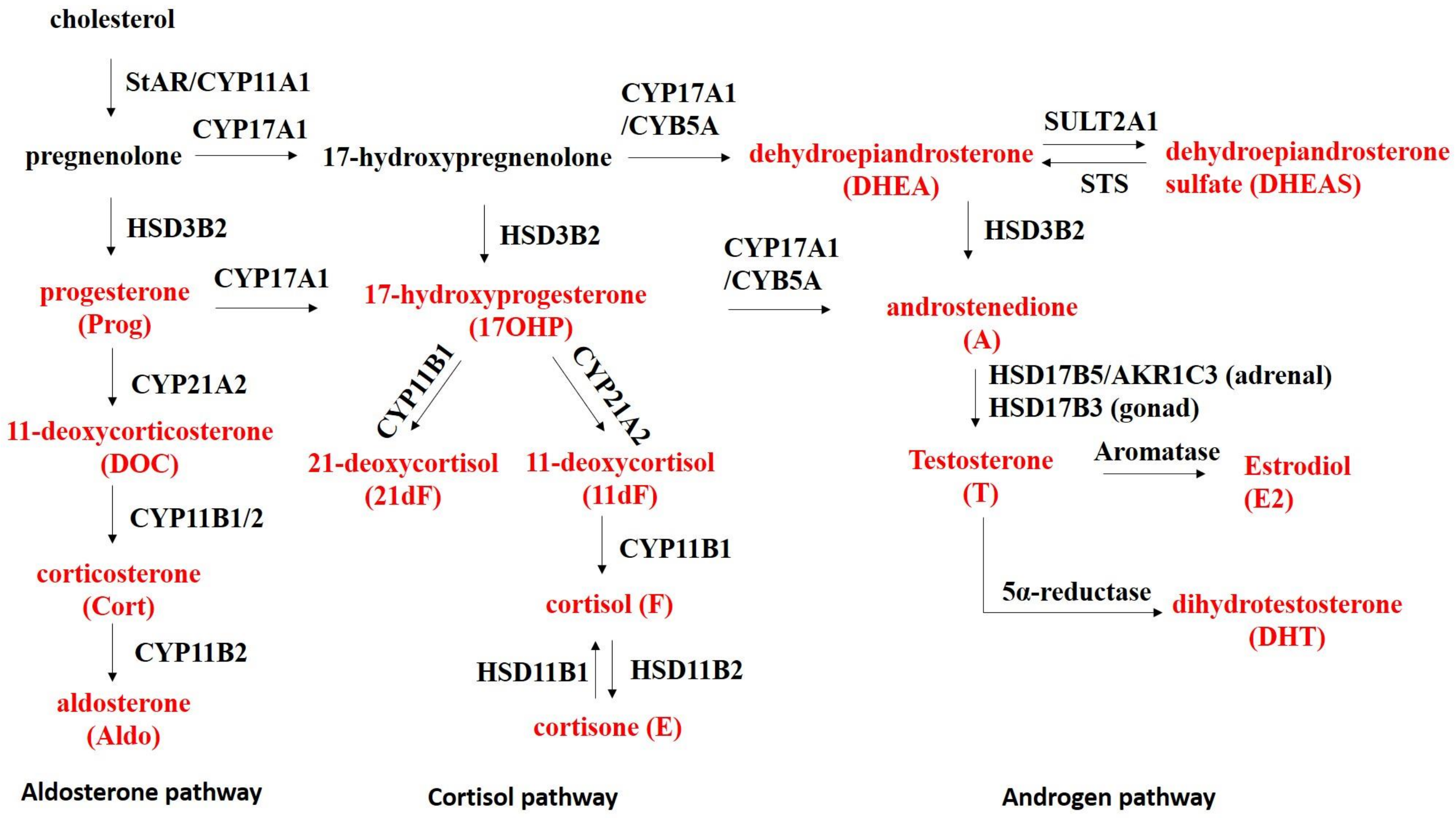

Figure S1. The pathway of adrenal steroidogenesis. The steroids in red are those we quantified.

2 Supplementary Tables

Table S1. Low limit of quantification and up limit of quantification of LC-MS/MS (ng/ml).

Pat. Nr.		Aldosterone	Cortisol	Cortisone	Corticosterone	11-deoxycortisol	21-deoxycortisol	DHEAS	Estradiol	Testosterone	Androstenedione	DHEA	DHT	17-hydroxyprogesterone	11-Deoxycorticosterone	Progesterone
1-9	LLOQ	0.014	1.52	0.148	0.175	0.03	0.027	24.4	0.026	0.005	0.022	0.229	0.042	0.04	0.023	0.03
	ULPQ	6	12000	80	2000	600	10	18000	10	24	600	120	3	600	120	45
10-16	LLOQ	0.028	1.225	0.102	0.08	0.144	0.042	14.4	0.004	0.01	0.032	0.213	0.081	0.015	0.008	0.035
	ULPQ	2.93	291	41	45.7	15.6	5.25	5944	5.14	12	13.9	58.2	1.43	14.7	2.95	25.7

Table S2. Demographic and clinical characteristics.

Patient No.	Sex	Age (at diagnosis)	Clinical presentation	LDDST [μg/dl]	UFC [μg/24h]	LNSLC [ng/ml]	ACTH [pg/ml]	Adrenal size on imaging				*ARMC5* Status
				Left		Right		
								WxDxH [cm]	Volume [ml]	WxDxH [cm]	Volume [ml]	
1	F	58	Overt CS	7.6	410	7.9	2.5	2.8x5.1x4.2	60	5.5x4.9x5.1	137.4	WT
2	F	60	Overt CS	5.6	169	2.3	9	2.6x5x4.2	54.6	2.3x4.3x5.6	55.4	WT
3	F	56	Overt CS	15.1	207	NA	<5	5x4.8x5.8	139.2	3.5x3.9x4.5	61.4	WT
4	M	48	Overt CS	33.7	847	10.3	3	8.2x10.5x9.9	852.4	4.7x3.9x6.3	115.5	MUT
5	F	58	Overt CS	4.1	222	3.3	<2	2.2x3.4x4	29.9	3.7x4.8x5.3	94.1	WT
6	F	38	Overt CS	14.5	324	5.9	3	4x1.3x4.4	22.9	1.4x3.5x4.9	24	WT
7	F	72	Overt CS	3.1	322	0.7	4	3x2.3x4.1	28.3	1.8x2.1x2.5	9.5	NA
8	F	50	Overt CS	13.2	NA	5.3	2	2.6x3.3x1.9	16.3	1.5x3.5x3.7	19.4	MUT
9	F	64	MACS	5.6	59	0.7	NA	3.2x2.8x3.7	33.2	2.4x2.6x3.9	24.3	WT
10	F	56	Overt CS	8.0	104	4.1	4	2.7x2.9x2.8	21.9	3.5x3.8x3.1	41.2	WT
11	F	58	Overt CS	4.3	151	5.6	<2	2.2x3.1		3.1x2.0		MUT
12	F	60	Overt CS	25.1	232	4.32	3	3.8x2x1.5	11.4	5.6x2.5x4.6	64.4	WT
13	F	69	Overt CS	12.5	80	4.8	<2	5.4x2.0x5.6	60.5	2.8x1.7x2.7	15.6	MUT
14	F	58	MACS	4.0	80	8.0	4	2.8x2.3x4.0	25.8	2.0x2.0x4.0	16	WT
15	F	55	MACS	2.7	64.8	4.6	7	2.9x1.7x3.7	18.2	2.5x0.7x1.8	3.2	NA
16	F	55	MACS	5.5	21.3	3.6	<5	3.8x4.7x3.4	60.7	2.0x2.3x3.1	14.3	MUT
17	F	77	Overt CS	8.8	36.8	1.6	6.3	3.3x2.6x3.0	25.7	2.8x1.9x2.7	14.4	MUT

Abbreviations: CS, Cushing's Syndrome; MACS, mild autonomous Cushing's Syndrome; ACTH: Adrenocorticotropic hormone (normal range: 10-50pg/ml); UFC: 24h urinary free cortisol measurement (normal range: 50-150μg/24h); LNSLC: late-night salivary cortisol (normal range: <1.5ng/ml); LDDST: Low Dose Dexamethasone Suppression Test (normal range: <1.8μg/dl).

Supplementary Table S3.1 Concentrations (ng/mL) of steroids of adrenal veins and peripheral vein quantified by LC-MS/MS.

Pat. Nr.	aldosterone			cortisol			cortisone			corticosterone			11-deoxycortisol		
	Left	Right	Peripheral	Left	Right	Peripheral	Left	Right	Peripheral	Left	Right	Peripheral	Left	Right	Peripheral
1	0.10	0.09	0.01	238	253	59	11	13	13	11.8	10.1	0.7	17.90	22.50	1.32
2	0.597	0.11	0.02	962	429	182	27	22	16	105.4	15.3	5.2	52.80	9.64	1.30
3	0.00	0.05	0.01	455	280	149	26	23	7	16.5	12.3	1.9	5.32	4.62	0.17
4	0.0011	0.0011	No Peak	640	377	168	30	35	11	183.2	102.0	17.0	22.20	17.30	1.75
5	1.52	0.0011	0.00	180	811	158	19	28	16	22.8	110.0	9.2	4.95	18.10	1.08
6	0.96	1.14	0.02	3000	3620	109	103	104	15	155.0	215.0	1.5	115.00	157.00	1.02
7	0.0136	0.114	No peak	383	10	102	20	47	15	13.6	72.1	1.3	3.87	16.70	0.29
8	1.76	0.79	0.0476	1600	404	104	93	38	18	115.0	34.4	2.0	23.00	4.93	0.40
9	0.02	0.20	No Peak	1280	245	135	48	18	19	111.4	38.4	2.3	40.80	12.20	0.75
10	0.07	0.60	0.08	292	1060	117	30	62	16	13.4	185.9	1.8	7.26	49.75	0.62
11	1.12	0.22	0.04	606	265	162	50	30	21	34.4	8.8	2.6	9.04	3.20	0.56
12	0.17	0.20	< 0.028	202	247	93	16	13	17	3.6	4.2	0.4	9.85	15.92	0.97
13	0.10	0.24	< 0.028	318	280	135	30	28	15	105.5	88.0	20.3	7.59	5.83	0.81
14	0.08	0.08	< 0.028	289	185	92	26	21	18	13.4	5.6	0.8	6.80	2.84	0.28
15	0.45	0.45	0.08	651	572	163	57	45	22	52.4	47.6	4.5	13.28	12.85	0.60
16	0.03	0.16	<0.01	333	216	86	30	20	13	30.6	18.1	2.4	4.96	3.96	0.37
17	1.49	0.124	0.0183	725	71	74	69	12	13	34.4	0.7	0.5	10.50	0.42	0.29

Supplementary Table 3.2 Concentrations (ng/mL) of steroids of adrenal veins and peripheral vein quantified by LC-MS/MS.

Pat. Nr.	21-deoxycortisol			DHEAS			estradiol			testosterone			androstenedione		
	Left	Right	Peripheral	Left	Right	Peripheral	Left	Right	Peripheral	Left	Right	Peripheral	Left	Right	Peripheral
1	1.30	1.09	0.09	2250	3010	2740	0.02	0.03	0.02	0.60	0.50	0.35	36.70	29.50	1.66
2	0.39	1.65	0.61	273	282	259	0.00	0.00	No Peak	0.30	0.22	0.12	20.70	13.00	2.23
3	1.82	1.36	0.22	440	511	369	0.09	0.08	0.07	0.10	0.13	0.05	4.47	4.82	0.76
4	0.24	10.90	2.55	72	69	62	0.01	0.01	0.01	1.61	1.51	2.10	1.98	3.08	0.29
5	2.60	11.90	1.00	470	560	459	0.00	0.00	No Peak	0.26	0.55	0.16	9.29	12.10	0.79
6	16.40	22.50	0.18	531	514	272	0.01	0.00	0.02	0.71	1.25	0.15	77.60	88.90	0.49
7	0.06	0.32	0.03	879	970	735	0.00	0.0014	0.00	0.26	0.45	0.20	7.57	26.2	0.50
8	11.80	3.91	0.25	115	150	119	0.05	0.07	0.0705	0.16	0.09	0.06	7.96	3.05	0.27
9	0.13	0.18	0.04	71	68	69	0.00	0.01	0.00	0.20	0.17	0.13	7.07	5.54	0.27
10	0.31	2.64	0.08	232	285	215	< 0.004	< 0.004	< 0.004	0.10	0.27	0.07	5.12	15.72	0.34
11	0.40	0.13	< 0.04	346	263	233	< 0.004	< 0.004	< 0.004	0.33	0.19	0.18	14.02	4.10	0.68
12	< 0.04	< 0.04	< 0.04	1136	911	908	< 0.004	0.03	< 0.004	0.45	0.34	0.22	7.31	14.23	1.01
13	0.34	0.21	0.05	48	45	41	< 0.004	< 0.004	< 0.004	0.03	0.03	0.03	0.83	1.14	0.08
14	0.18	0.06	< 0.04	293	257	239	0.12	0.02	0.06	0.14	0.07	0.07	5.87	2.29	0.31
15	0.08	< 0.04	< 0.04	238	209	183	0.04	< 0.004	0.01	0.39	0.40	0.20	15.09	10.24	0.58
16	0.05	0.12	0.02	<100	<100	<100	<0.03	<0.03	<0.03	0.12	0.08	0.05	3.75	4.58	0.17
17	0.28	0.02	0.03	475	291	335	<0.03	<0.03	<0.03	0.27	0.17	0.11	12.50	0.42	0.34

Supplementary Table 3.3 Concentrations (ng/mL) of steroids of adrenal veins and peripheral vein quantified by LC-MS/MS.

Pat. Nr.	DHEA			dihydrotestosterone			17-hydroxyprogesterone			11-deoxycorticosterone			progesterone		
	Left	Right	Peripheral	Left	Right	Peripheral	Left	Right	Peripheral	Left	Right	Peripheral	Left	Right	Peripheral
1	12.90	6.81	1.82	0.21	0.29	0.29	4.38	3.00	0.31	0.76	0.87	0.07	0.34	0.24	0.05
2	7.48	8.04	0.86	0.26	0.45	0.40	24.90	2.87	0.15	7.84	0.64	0.10	4.86	0.26	0.01
3	1.99	4.31	0.26	0.53	0.31	0.24	2.18	2.37	0.19	0.34	0.27	0.00	0.11	0.10	No Peak
4	1.04	0.83	0.49	0.35	0.33	0.30	3.53	5.98	0.69	5.29	4.02	0.44	1.75	2.08	0.09
5	9.89	16.40	3.78	0.24	0.24	0.24	4.28	12.00	0.35	0.99	2.04	0.14	0.47	2.26	0.05
6	20.60	20.60	0.33	0.04	0.04	0.04	40.20	46.30	0.25	7.37	11.10	0.07	4.97	7.05	0.40
7	10.70	23.10	0.98	0.02	0.00	0.00	2.81	11.00	0.29	0.23	1.35	0.06	0.18	0.93	0.05
8	2.21	1.75	0.48	0.02	No Peak	0.14	7.60	2.03	0.40	1.82	0.56	0.04	1.29	1.34	0.83
9	1.39	3.64	0.12	0.12	0.17	0.12	5.18	4.27	0.11	3.39	4.24	0.15	1.32	2.32	0.00
10	7.14	20.69	1.17	< 0.08	< 0.08	< 0.08	3.60	40.71	0.25	0.44	14.71	0.04	0.11	17.50	< 0.04
11	12.10	3.86	0.71	< 0.08	< 0.08	< 0.08	6.72	2.01	0.38	0.77	0.26	0.04	0.32	< 0.04	< 0.04
12	3.48	3.29	0.85	< 0.08	< 0.08	< 0.08	1.58	2.92	0.18	0.37	0.44	0.04	< 0.04	< 0.04	< 0.04
13	0.25	0.79	< 0.21	< 0.08	< 0.08	< 0.08	3.26	2.27	0.23	2.42	1.41	0.27	1.33	0.72	0.07
14	5.90	2.92	0.56	< 0.08	< 0.08	< 0.08	3.34	1.39	0.16	0.36	0.15	0.03	< 0.04	< 0.04	< 0.04
15	14.19	11.97	1.42	< 0.08	< 0.08	< 0.08	4.82	4.95	0.19	1.13	1.40	0.07	0.40	0.53	< 0.04
16	1.76	3.12	0.16	<0.17	<0.17	<0.17	2.21	2.30	0.12	<0.10	<0.09	<0.07	<0.08	<0.07	<0.05
17	12.4	0.84	0.64	<0.17	<0.17	<0.17	8.10	0.19	0.10	1.16	<0.07	<0.07	0.73	<0.05	<0.05

Table S3 Comparison of clinical parameters based on ARMC5 status

clinical parameters	wild-type *ARMC5*	mutant *ARMC5*	*p* value
AVol (ml)	75.8[52.2, 160.7]	75.0[37.9, 522.0]	0.61
LDDST [µg/dl]	7.6[4.9, 14.8]	10.7[5.2, 18.3]	0.67
UFC [µg/24h]	207[92, 278]	80.0[29.1, 499.0]	0.25
LNSLC [ng/ml]	4.2[2.6, 7.4]	5.1[3.1, 6.8]	0.75
ACTH [pg/ml]	3.0[2.5, 4.0]	2.3[1.0, 3.8]	0.26

AVol, adrenal volume; LDDST: Low Dose Dexamethasone Suppression Test; UFC: 24h urinary free cortisol measurement; LNSLC: late-night salivary cortisol; ACTH: Adrenocorticotropic hormone.
